# Microstructure Evolution and Fracture Mode of Laser Welding–Brazing DP780 Steel-5754 Aluminum Alloy Joints with Various Laser Spot Positions

**DOI:** 10.3390/ma18122676

**Published:** 2025-06-06

**Authors:** Bolong Li, Jiayi Zhou, Rongxun Hu, Hua Pan, Tianhai Wu, Yulai Gao

**Affiliations:** 1 State Key Laboratory of Advanced Special Steel, School of Materials Science and Engineering, Shanghai University, Shanghai 200444, China; libolong_111@shu.edu.cn (B.L.); zhoujy00@shu.edu.cn (J.Z.); rongxunhu@shu.edu.cn (R.H.); 2 Automobile Steel Research Institute, R&D Center, Baoshan Iron & Steel Co., Ltd., Shanghai 201900, China; hpan@baosteel.com

**Keywords:** steel–Al joint, laser welding–brazing, intermetallic compound, laser spot position, fracture mode

## Abstract

Joining steel and Al alloys can fully utilize their advantages for both base metals (BMs) and optimize automobile structures. In this study, the laser welding–brazing technique was utilized to join DP780 steel and aluminum alloy 5754 (AA5754). The mechanical properties, microstructure, and fracture locations of steel–Al joints prepared using different laser spot positions were comparatively investigated. As the proportion of the laser spot on the steel BM increased from 50% to 90%, the tensile–shear strength of the steel–Al welded joint rose from 169 MPa to 241 MPa. Meanwhile, the fracture location of the joint shifted from the interface to the BM of the aluminum alloy. The change in the laser spot position could dramatically affect the interfacial microstructure and fracture mode of the steel–Al joint. When the proportion of the laser spot on the steel BM was relatively small (50%), the growth of intermetallic compounds (IMCs) was inhibited. The metallurgical bonding effect at the steel–Al interface was poor. In this case, the interfacial zone became the primary path for the crack propagation. Thus, interface failure became the dominant failure mode of the steel–Al joint. On the contrary, metallurgical bonding at the interface was remarkably improved as the proportion of the laser spot on the BM of the steel increased (to 90%). It was determined that the IMCs could effectively hinder the propagation of cracks along the interface. Eventually, the joint fractured in the Al alloy’s BM, resulting in a qualified steel–Al joint.

## 1. Introduction

With the intensification of the energy crisis and the increasing strictness of environmental protection regulations, lightweight has become an important development direction [1,2,3]. Lowering vehicle weight can significantly lower energy consumption [4]. In addition, lightweighting can enhance the acceleration, braking, and other performance metrics of the car, resulting in a better driving experience for drivers [5]. Among numerous lightweight materials, aluminum alloy is extensively employed in automotive production owing to its low density and good corrosion resistance [6]. However, it is not practical to manufacture cars entirely from aluminum alloy [6,7]. For some components with high strength requirements, steel is still an indispensable material [8]. Combining steel with Al alloys and maximizing the advantages of both materials has become a preferred way to achieve automotive lightweight [9,10,11].

Due to the significant differences in physical and chemical properties between steel and aluminum alloy, joining dissimilar steel–aluminum materials faces numerous difficulties [5,12,13]. To prepare qualified steel–Al welded joints, researchers have carried out extensive studies on the welding techniques of dissimilar steel–aluminum metals [14]. Xu et al. [15] utilized the resistance spot welding technique to join Al alloy 6061 and low-alloy carbon steel. With the increased welding current, welding time, and electrode pressure, the strength of the resistance spot welding specimens exhibited the tendency to rise initially and then decline. With a welding current of 16 kA, a welding time of 300 ms, and an electrode pressure of 3 kN, the joint strength reached its maximum value of 2.24 kN. Mortazavi et al. [16] found that the size of the nugget zone became larger with the increasing welding current. The fracture mode of the resistance spot welded joint gradually changed from interfacial fracture to pullout fracture. According to Zhou et al. [17], the addition of the CoCrFeNiMn high-entropy alloy interlayer could slow down the growth of intermetallic compounds and increase nugget zone diameter. As a result, a high-quality resistance spot welding steel–Al joint was obtained. Geng et al. [18] prepared steel–Al lap joints via friction stir welding. The number of pores in the interfacial region first decreased and then increased with the tool rotation speed. The thickness of the IMCs continuously increased. Zhang et al. [19] proposed an innovative friction stir welding process to reduce the wear of the tool. The tool did not contact the steel base metal (BM). In this case, the stirring needle could be fully plunged into the thickened Al alloy BM. Geng et al. [20] utilized the magnetic pulse welding technique to fabricate a steel–Al composite structure. The results revealed that with the appropriate increase in the discharge energy, the weld seam area gradually increased, improving joint strength. Yu et al. [21] fabricated an inclined-wall field shaper. When it was not used, there were many defects and a relatively thick transition zone at the interface of the magnetic pulse welding specimen. When the inclined-wall field shaper was adopted, the interface morphology changed from flat to wavy. Meanwhile, the defects were reduced, and the transition zone became thinner. Chen et al. [22] employed the explosive welding technique to fabricate the dissimilar steel–Al welded joint. Inserting a SUS821L1 stainless steel interlayer between the BMs could reduce kinetic energy dissipation and lower the temperature at the interface while welding dissimilar metals. As a result, high-quality welding between steel and Al alloy was achieved. Torkamany et al. [23] fabricated steel–aluminum dissimilar joints via laser welding. The IMC layer thickened with the laser power, and the pulse duration increased. Meanwhile, the probability of defects also increased.

As an advanced welding technique with broad prospects in preparing steel–aluminum composite structures, laser welding–brazing has attracted extensive attention. The growth and distribution of interfacial IMCs are closely related to the properties of laser welding–brazing steel–Al joints [24]. Li et al. [25] used four laser powers (1800 W, 2200 W, 2500 W and 3000 W), respectively, to fabricate steel–Al butt joints. Overly low laser power (1800 W) severely restricted the growth of IMCs. On the contrary, high laser powers (2500 W and 3000 W) can lead to the excessive growth of IMCs. The IMCs formed in these two conditions deteriorated the bonding quality of the steel–Al interface. Moderate laser power (2200 W) can promote the normal growth of IMCs, thereby improving the interfacial microstructure to successfully resist crack propagation and achieve the optimized mechanical properties of the joint. Dharmendra et al. [26] fabricated DP600 galvanized steel and 6016 Al alloy lap joints using the laser welding–brazing technique. When the IMC layer was too thin or thick, the welded joints were prone to brittle fracture in the interfacial region. Nevertheless, the appropriate regulation of the welding speed could optimize the thickness of the IMC layer. As a result, the fracture positions of the welded joints were concentrated in the BM of the Al alloy. Ding et al. [27] pointed out that the cracks easily propagated along the coarse IMC layer during the tensile test. In contrast, a uniformly and continuously distributed IMC layer could effectively hinder the crack propagation. A uniformly distributed interfacial IMC layer was obtained by adjusting the welding heat input, which was beneficial to the improvement of the joint mechanical properties. Yan et al. [28] regulated the interfacial metallurgical reaction of the laser welding–brazing steel–Al joint by applying a magnetic field. The results showed that applying the magnetic field could promote the moderate growth and reasonable distribution of IMCs, thus enhancing the interfacial bonding strength.

In summary, the growth and distribution of the interfacial IMCs could significantly affect the properties of laser welding–brazing steel–Al joints. Optimizing the welding parameters was anticipated to promote the moderate growth and reasonable distribution of the IMCs, thereby improving the joint properties [29]. Nevertheless, little information is available regarding the effect of laser spot position on the interfacial microstructure and mechanical properties of joints [30,31,32]. In the present study, two laser spot positions (50/50 and 90/10) were chosen to prepare the lapped joints of DP780 steel and aluminum alloy 5754 (AA5754). Moreover, the microstructure evolution and fracture mode of the laser welding–brazing joint with various laser spot positions were systematically investigated and discussed.

## 2. Experimental Procedures

**Figure 1:**
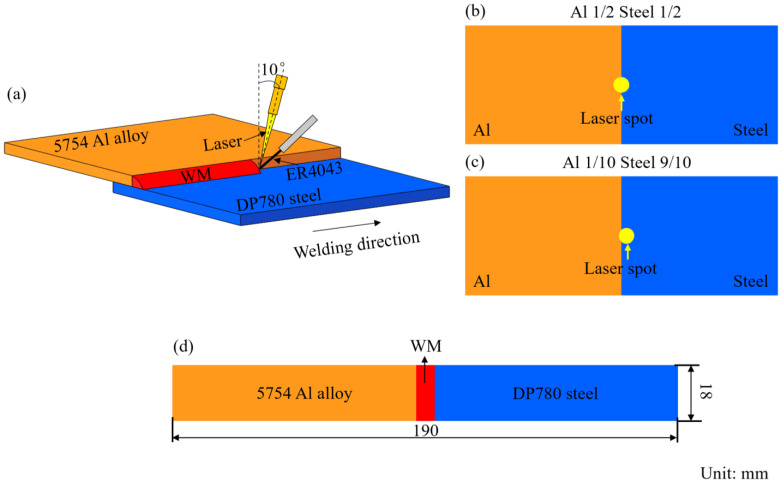
Schematic of the laser brazing–welding process, laser spot position, and tensile–shear specimen: (**a**) the laser brazing–welding process; (**b**,**c**) the laser spot position; (**d**) the tensile–shear specimen.

Hot-dip galvanized DP780 steel was used as the steel BM. Zn coating could improve the oxidation resistance and corrosion resistance of the steel plate [33,34]. AA5754 was employed as the BM of the Al alloy. It had good corrosion resistance and weldability [35]. An ER4043 (AlSi5) wire with a Si content of 5 wt.% was selected as the filler material. The Si element could reduce the brittleness of the steel–Al interfacial zone [36]. The chemical composition of the BMs and the filler material is listed in Table 1. To clarify the influence of the laser spot position on the mechanical properties, microstructure, and fracture location of the steel–Al welded joint, two laser spot positions were chosen, namely 50/50 (steel–Al) and 90/10 (steel–Al). The position 50/50 (steel–Al) indicated that the proportion of the laser spot on both the steel BM and the Al alloy BM was 50%. The position 90/10 (steel–Al) demonstrated that the laser spot accounted for 90% on the steel BM and 10% on the Al alloy BM. Other welding parameters (such as laser power, welding speed, etc.) were kept unchanged. The detailed welding parameters employed in this investigation are listed in Table 2. Figure 1a–c show the sketch of the welding process and laser spot position.

Tensile–shear tests were conducted on steel–Al welded joints prepared with different laser spot positions using a material testing machine (Instron 5581, Instron, Norwood, MA, USA). The focus was on investigating the effect of the laser spot position on the tensile–shear strength of the steel–Al joints. The plates of the steel and Al alloy base metals were cut using a shearing machine. The dimensions of the tensile–shear specimens were determined according to GB/T 26957-2022 [37]. Figure 1d displays the schematic of the tensile–shear specimen. After the tensile–shear tests, microstructure analysis was carried out on the steel–Al welded joints. These specimens were ground, polished, and etched. The etchants selected were nitric acid alcohol (4% HNO_3_ + C_2_H_5_OH with the volume proportion of 5:1) and Keller’s reagent (HF + HCl + HNO_3_ + H_2_O with the volume proportion of 1:1:1:6). The former was used to etch the microstructure of the steel BM. The latter was employed to reveal the microstructure of the Al alloy BM and the weld seam. The microstructure of the steel–Al joints was observed using an optical microscope (OM, Zeiss Axioscope 5, Zeiss, Oberkochen, Germany) and scanning electron microscope (SEM, VEGA3 SBU—EasyProbe, TESCAN, Brno, Czech Republic). The elemental composition of the IMCs was detected by an energy dispersive spectrometer (EDS, Bruker Xflash, Bruker, Billerica, MA, USA). A systematic study was carried out on the morphology differences in interfacial IMCs of the specimens prepared with different laser spot positions.

**Table 1:** The chemical composition of the base metals (BMs) and the filler material (wt.%). Reprinted from Ref. [38].

Elements	Si	Mn	Ti	Cu	Mg	Cr	Zn	C	P+S	Fe	Al
5754	0.4	0.5	-	0.1	3.5	0.3	0.2	-	-	0.4	Bal.
DP780	≤0.9	≤2.5	-	-	-	-	-	≤0.18	≤0.06	Bal.	-
ER4043	5	<0.05	<0.15	<0.05	-	<0.05	<0.1	-	-	<0.6	Bal.

**Table 2:** Parameters employed for the laser welding–brazing process.

Welding Parameters	#1	#2
Laser power, W	1800	
Welding speed, m/min	0.36	
Wire feed speed, m/min	3.0	
Laser tilt angle	10°	
Position of laser spot	50/50 (steel–Al)	90/10 (steel–Al)

## 3. Results and Discussion

**Figure 2:**
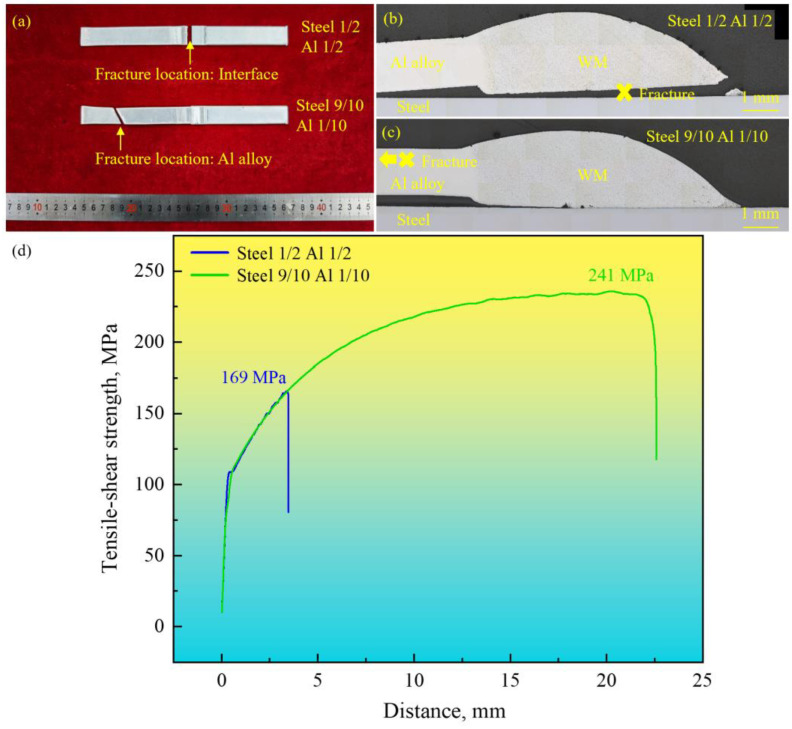
The tensile–shear test results of the specimens prepared with various laser spot positions: (**a**) the appearances of the specimens after the tensile–shear test, (**b**,**c**) the overall morphology of the specimens after the tensile–shear test, and (**d**) tensile–shear curves.

Figure 2 displays the tensile–shear test results of steel–Al welded joints prepared with different laser spot positions. When the laser spot position was 50/50 (steel–Al), the joint fractured at the interface (see Figure 2b). The corresponding tensile–shear strength was only 169 MPa (see Figure 2d). When the laser spot position was 90/10 (steel–Al), the joint failed in the Al alloy (see Figure 2c). The specimen strength increased to 241 MPa (see Figure 2d). The joint property could be dramatically improved as the proportion of the laser spot on the steel BM increased from 50% to 90% according to the test results. Compared with other investigations with joint strengths of 210 MPa, 142 MPa, and 227 MPa [24,25,26], the strength of 241 MPa of the steel–Al joint prepared with the laser spot position of 90/10 (steel–Al) was satisfactory in this work.

**Figure 3:**
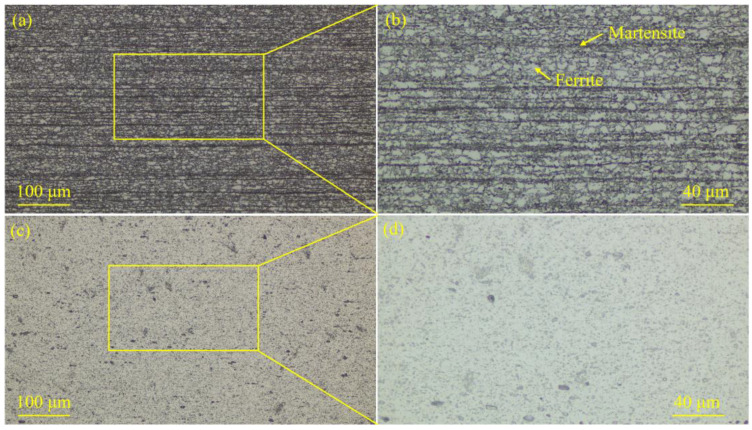
The metallographic microstructure of the steel BM (DP780 steel) and the Al alloy BM (5754 Al alloy): (**a**,**b**) the metallographic microstructure of the steel BM; (**c**,**d**) the metallographic microstructure of the Al alloy BM.

**Figure 4:**
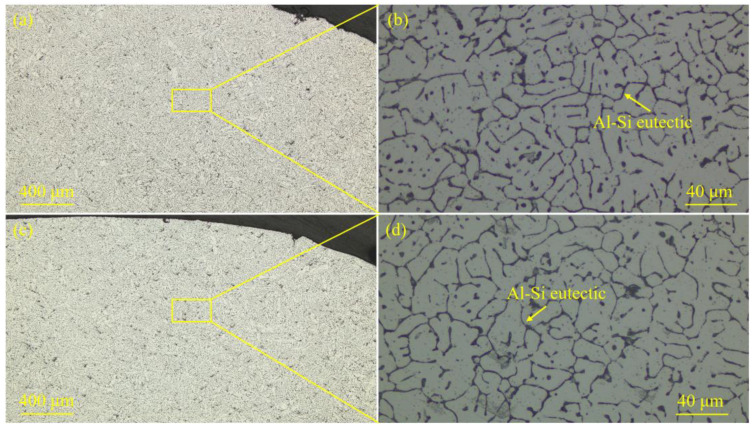
The metallographic microstructure of the weld seam of the steel–Al welded joints prepared with different laser spot positions: (**a**,**b**) the metallographic microstructure of the weld seam of the steel–Al welded joint corresponding to the laser spot position of 50/50 (steel–Al); (**c**,**d**) the metallographic microstructure of the weld seam of the steel–Al welded joint corresponding to the laser spot position of 90/10 (steel–Al).

The microstructure of DP780 steel and 5754 Al alloy is presented in Figure 3. Figure 4 displays the weld seam microstructure of the specimens prepared with different laser spot positions. The microstructure of DP780 steel was mainly composed of ferrite and martensite (see Figure 3a,b). The 5754 Al alloy microstructure was mainly α-Al (see Figure 3c,d). During the laser welding–brazing, the Si element was mixed into the molten pool through the ER4043 (AlSi5) filler metal. Therefore, the Al-Si eutectic distributed along the grain boundaries of α-Al could be clearly observed in the weld seam (see Figure 4).

**Figure 5:**
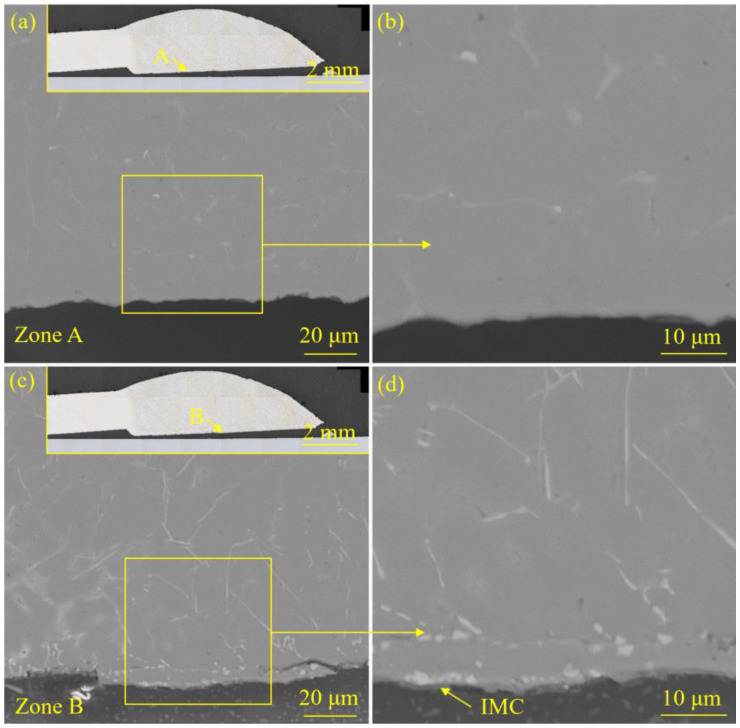
The interface morphology of the welded joint prepared with the laser spot position of 50/50 (steel–Al): (**a**) the microstructure details of zone A, (**b**) the local enlarged image of zone A, (**c**) the microstructure details of zone B, and (**d**) the local enlarged image of zone B.

**Figure 6:**
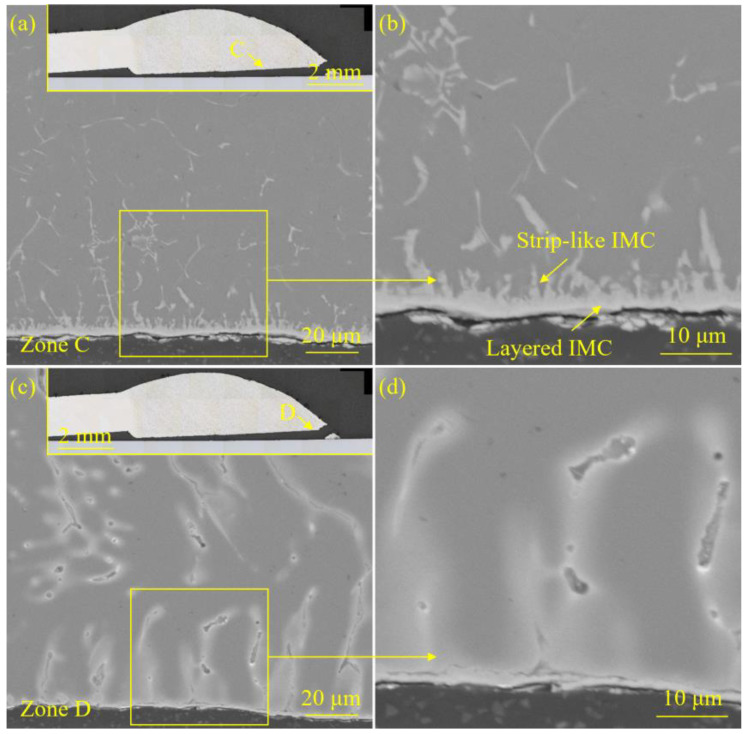
The interface morphology of the welded joint prepared with the laser spot position of 50/50 (steel–Al): (**a**) the microstructure details of zone C, (**b**) the local enlarged image of zone C, (**c**) the microstructure details of zone D, and (**d**) the local enlarged image of zone D.

**Figure 7:**
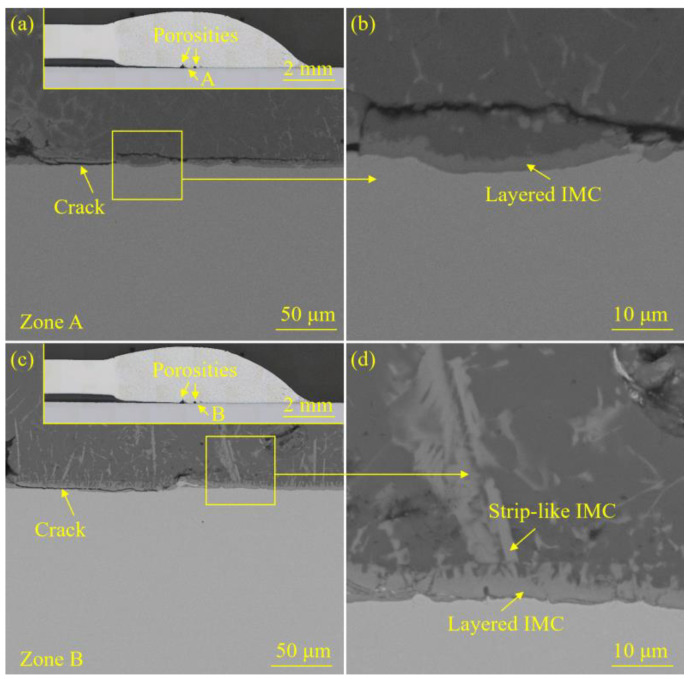
The interface morphology of the welded joint prepared with the laser spot position of 90/10 (steel–Al): (**a**) the microstructure details of zone A, (**b**) the local enlarged image of zone A, (**c**) the microstructure details of zone B, and (**d**) the local enlarged image of zone B.

**Figure 8:**
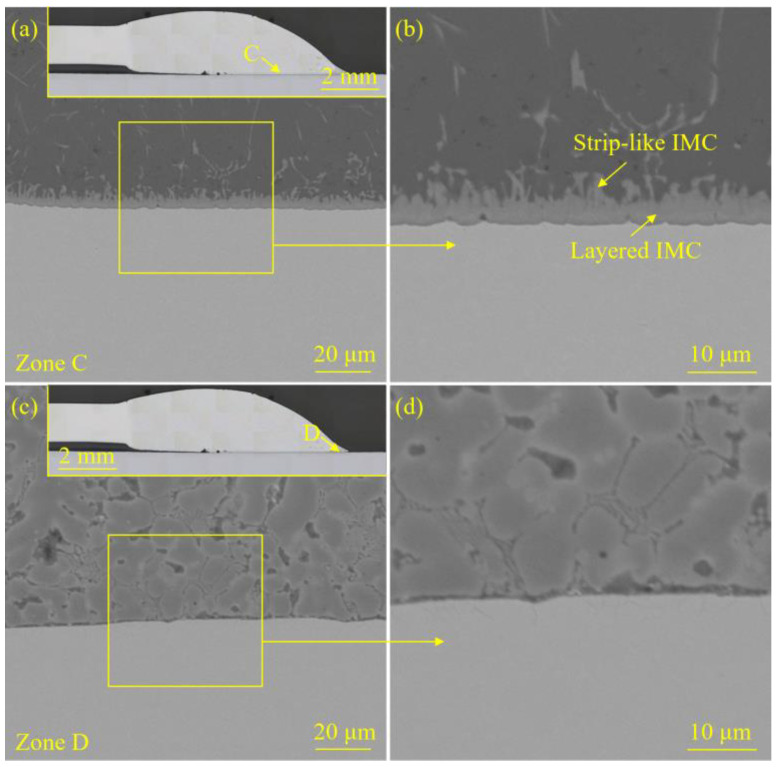
The interface morphology of the welded joint prepared with the laser spot position of 90/10 (steel–Al): (**a**) the microstructure details of zone C, (**b**) the local enlarged image of zone C, (**c**) the microstructure details of zone D, and (**d**) the local enlarged image of zone D.

Figure 5 and Figure 6 exhibited the interface morphology of the welded joint prepared with the laser spot position of 50/50 (steel–Al). Figure 7 and Figure 8 display the interface morphology of the welded joint prepared with the laser spot position of 90/10 (steel–Al). To shed much light on the IMC characteristics of the laser welding–brazing joint, four different zones (zones A, B, C, and D) at the interface were observed, respectively.

For the specimen corresponding to the laser spot position of 50/50 (steel–Al), no obvious IMC was detected in zones A and D (see Figure 5a,b and Figure 6c,d). Discontinuous and extremely thin IMCs were formed in zone B (see Figure 5c,d). Two forms of interfacial IMCs were observed in zone C: layered and strip-like (see Figure 6a,b). During the welding process, the uneven heating input in the interfacial zone led to different IMC growth.

For the specimen corresponding to the laser spot position of 90/10 (steel–Al), there were porosity defects near zones A and B (see the insets in Figure 7a,c). The cracks initiated at the porosities and subsequently propagated along the interface. Finally, the crack propagation was successfully hindered by the IMCs (see Figure 7a,c). Interestingly, the porosities and cracks that appeared at the interface did not cause too much adverse impact on the mechanical properties of the joint. There were certain differences in the growth conditions of the IMCs in various interfacial zones. The thinner layered IMC was detected in zone A (see Figure 7a,b). Layered IMC and strip-like IMC were observed in zones B and C (see Figure 7c,d and Figure 8a,b). Compared with zone C, the IMCs in zone B grew faster and became coarser. The growth of IMC in zone D was thus inhibited, so no obvious IMC could be found in this zone (see Figure 8c,d).

The change in the position of the laser spot significantly affected the interfacial microstructure and the fracture location of the steel–Al welded joint. When the proportion of the laser spot on the steel BM was relatively small (50%), the growth of the IMCs was severely inhibited. In this case, the effect of interfacial metallurgical bonding of the steel–Al joint was poor. The interface became the zone where cracks easily propagated. Eventually, the joint prepared with the laser spot position of 50% on the steel and 50% on the Al fractured at the interface. As the proportion of the laser spot on the steel BM increased from 50% to 90%, the steel experienced more heat input. This change in heat transfer promoted the wetting and spreading of the molten Al alloy and filler material on the steel surface [30], accelerating the diffusion and reaction of Fe, Al, and Si atoms at the steel–Al interface. Consequently, the growth of IMCs was promoted, leading to significant improvement in the interfacial metallurgical bonding and a substantial increase in the joint strength. Although cracks initiated in the interfacial zone during the tensile–shear test, the IMCs at the interface could effectively hinder the further propagation of the cracks along the interface [39]. Therefore, the joint prepared with the laser spot position of 90% on the steel and 10% on the Al failed at the aluminum alloy BM.

**Figure 9:**
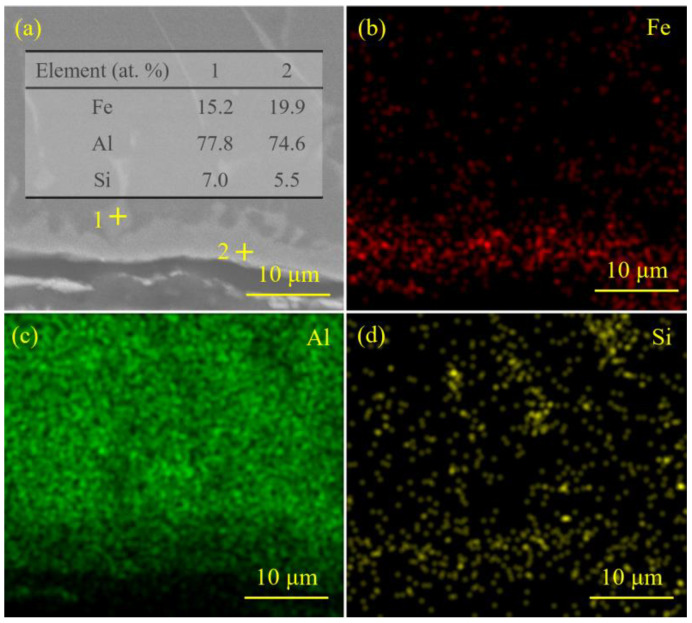
The elemental analysis results of the interfacial zone corresponding to the laser spot position of 50/50 (steel–Al): (**a**) the SEM image of the interfacial zone, 1+ and 2+ in (**a**) represent the test points 1 and 2 of EDS point scanning, respectively, the results of the EDS point scanning of the IMCs at the interface are listed in the inserted table, (**b**–**d**) the results of the EDS mapping of the interfacial zone.

Figure 9 demonstrates the elemental analysis results of the interfacial zone corresponding to the laser spot position of 50/50 (steel–Al). Based on the results of EDS point scanning and mapping, the IMCs at the interface were composed of elements Fe, Al, and Si. The chemical potential of Si atoms at the steel–Al interface was lower than that in the weld seam and the steel substrate in the Fe-Al-Si ternary system [36]. Therefore, Si atoms tended to diffuse toward the steel–Al interface, thereby reacting with Fe and Al in the interfacial region to form ternary Fe-Al-Si IMCs.

**Figure 10:**
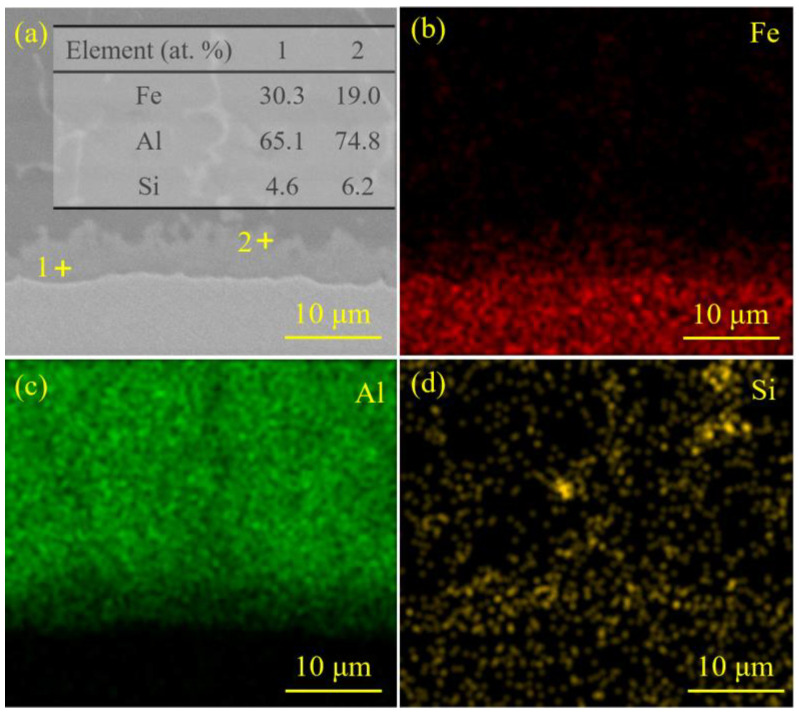
The elemental analysis results of the interfacial zone corresponding to the laser spot position of 90/10 (steel–Al): (**a**) the SEM image of the interfacial zone, 1+ and 2+ in (**a**) represent the test points 1 and 2 of EDS point scanning, respectively, the results of the EDS point scanning of the IMCs at the interface are listed in the inserted table, (**b**–**d**) the results of the EDS mapping of the interfacial zone.

Figure 10 demonstrates the elemental analysis results of the interfacial zone corresponding to the laser spot position of 90/10 (steel–Al). Similarly to the steel–Al joint with the laser spot position of 50/50 (steel–Al), the interfacial zone of the steel–Al joint with the laser spot position of 90/10 (steel–aluminum) formed the ternary Fe-Al-Si IMCs. During the welding process, Si atoms were prone to accumulate at the steel–Al interface, which increased the growth activation energies of the binary Fe-Al IMCs. Eventually, the growth of hard and brittle Fe-Al IMCs was effectively inhibited. The ternary Fe-Al-Si IMCs with appropriate thickness could significantly improve the toughness of the interfacial zone and its ability to resist the crack propagation [40,41]. The formation of this interfacial microstructure was beneficial to the enhancement of the steel–Al welded joint strength.

## 4. Conclusions

In the present study, the laser welding–brazing technique was employed to prepare steel–Al welded joints. A steel–Al welded joint with excellent mechanical properties was obtained by adjusting the position of the laser spot. Moreover, the effect of the laser spot position on the mechanical properties and the evolution of the microstructure of the steel–Al welded joint was comparatively analyzed. The main conclusions are listed as follows:

(1) The microstructure of the steel base metal (BM) was mainly composed of ferrite and martensite. The microstructure of the Al alloy BM was mainly α-Al. The weld seam microstructure of the steel–Al welded joints prepared with two laser spot positions was both α-Al matrix and Al-Si eutectic precipitated phases. The IMCs at the interface were all composed of elements Fe, Al, and Si.
(2) As the proportion of the laser spot on the steel BM increased from 50% to 90%, the tensile–shear strength of the steel–Al welding–brazing joint rose from 169 MPa to 241 MPa. The fracture location of the joint changed from the interface to the BM of the Al alloy, implying that the departure of the laser spot from the Al BM was favorable to prepare a qualified steel–Al welding–brazing joint.
(3) A relatively small proportion (50%) of the laser spot on the steel BM could severely restrict the growth of intermetallic compounds (IMCs) and deteriorate the effect of interfacial metallurgical bonding of the steel–Al joint. In this case, the interfacial zone became the primary path for the crack propagation. The interface failure became the dominant failure mode of the steel–Al joint. As the proportion of the laser spot on the steel BM increased (to 90%), the metallurgical bonding at the interface was remarkably improved. The IMCs could effectively hinder the propagation of cracks along the interface. Eventually, the joint fractured at the BM of the Al alloy.
(4) It is necessary to clarify the synergistic effects of various process parameters (laser spot position, welding speed, and laser power, etc.) on joint quality in future research. Moreover, the process window should be determined to achieve the qualified welding–brazing steel–Al joints in industry.

## Data Availability

The original contributions presented in this study are included in the article. Further inquiries can be directed to the corresponding authors.
